# Contemporary Treatment and Outcomes of High Surgical Risk Mitral Regurgitation

**DOI:** 10.3390/jcm12082978

**Published:** 2023-04-19

**Authors:** Mitsumasa Sudo, Vivian Vij, Nihal Wilde, Tetsu Tanaka, Johanna Vogelhuber, Miriam Silaschi, Marcel Weber, Farhad Bakhtiary, Georg Nickenig, Sebastian Zimmer, Atsushi Sugiura

**Affiliations:** 1 Heart Center Bonn, Department of Internal Medicine II, University Hospital Bonn, 53179 Bonn, Germany; 2 Department of Cardiology, Division of Medicine, Nihon University School of Medicine, Tokyo 173-8610, Japan; 3 Heart Center Bonn, Department of Cardiac Surgery, University Hospital Bonn, 53179 Bonn, Germany

**Keywords:** mitral regurgitation, mitral valve intervention, optimal medical therapy alone, contemporary management

## Abstract

Before the development of transcatheter interventions, patients with mitral regurgitation (MR) and high surgical risk were often conservatively treated and subject to poor prognoses. We aimed to assess the therapeutic approaches and outcomes in the contemporary era. The study participants were consecutive high-risk MR patients from April 2019 to October 2021. Among the 305 patients analyzed, 274 (89.8%) underwent mitral valve interventions, whereas 31 (10.2%) received medical therapy alone. Of the interventions, transcatheter edge-to-edge mitral repair (TEER) was the most frequent (82.0% of overall), followed by transcatheter mitral valve replacement (TMVR) (4.6%). In patients treated with medical therapy alone, non-optimal morphologies for TEER and TMVR were shown in 87.1% and 65.0%, respectively. Patients undergoing mitral valve interventions experienced less frequent heart failure (HF) rehospitalization compared to those with medical therapy alone (18.2% vs. 42.0%, *p* < 0.01). Mitral valve intervention was associated with a lower risk of HF rehospitalization (HR 0.36 [0.18–0.74]) and an improved New York Heart Association class (*p* < 0.01). Most high-risk MR patients can be treated with mitral valve interventions. However, approximately 10% remained on medical therapy alone and were considered as unsuitable for current transcatheter technologies. Mitral valve intervention was associated with a lower risk of HF rehospitalization and improved functional status.

## 1. Introduction

Mitral regurgitation (MR) is a highly prevalent valvular disease, significantly contributing to the risk of mortality and heart failure [1]. However, formerly, only a minority of patients with significant MR were able to undergo mitral surgery due to a high burden of comorbidities [2]. Recently, less invasive transcatheter intervention technologies have been developed and applied to treat MR patients with high surgical risk [3]. Transcatheter edge-to-edge repair (TEER) is one of the most widespread transcatheter techniques, showing its safety, feasibility, and efficacy on the clinical prognosis of patients with MR [4,5]. Due to the high demand for minimally invasive procedures in high-risk patients, the number of transcatheter procedures has been increasing in the United States and European countries [6]. However, given the complexity of mitral valve anatomy, not all patients are suitable for TEER [3]. Other transcatheter technologies, such as transcatheter mitral valve replacement (TMVR), have also shown remarkable progress and are already commercially available in European countries [7,8], which can allow physicians to provide a wide array of potential treatment options for each individual. Nonetheless, the current management of therapeutic approaches for patients with MR and high surgical risk remains modestly unknown. Knowing contemporary treatment and the outcomes of MR will be essential in proceeding with the development of clinical practice. In this context, we assessed the clinical characteristics of the treatment and outcomes of significant MR in patients with high surgical risk in the contemporary era.

## 2. Methods

### 2.1. Study Population

We retrospectively reviewed consecutive patients with moderate-to-severe or severe MR who were deemed at high surgical risk, and we discussed transcatheter mitral valve treatment at the local interdisciplinary heart team conference at the University Hospital Bonn from April 2019 to October 2021. A patient’s surgical risk was not merely defined by calculating risk scores but was rather evaluated by the local interdisciplinary heart team incorporating additional non-numeric factors such as frailty, nutritional status, mental illness, or procedure-specific impediment [9]. We excluded patients requiring revascularization of coronary artery disease, cardiac resynchronization therapy, or interventions for other valve diseases before mitral valve interventions [10]. Patients who died before performing mitral valve treatment were excluded from the present analysis. This study was conducted according to the guidelines detailed in the Declaration of Helsinki and with the approval of the Ethics Committee, Faculty of Medicine, University of Bonn (187/10). All patients provided informed consent to the data acquisition.

### 2.2. Echocardiographic Assessment

Transthoracic and transesophageal echocardiography was routinely performed before the heart team conference. Two dedicated physicians assessed the obtained images according to the current guidelines of the American Society of Echocardiography and the European Society of Echocardiography [10,11]. Based on comprehensive assessments by quantitative and qualitative measurements, the severity of MR was graded as follows: 0, none; 1+, mild; 2+, moderate; 3+, moderate-to-severe; and 4+, severe. Transesophageal echocardiographic images were assessed for the morphological features of the mitral valve. The etiology of MR was determined to be either primary or secondary in origin, or recurrent. In patients treated with medical therapy alone, the morphological characteristics of the mitral valve were determined according to German criteria [12]. Additionally, in the patients with secondary MR, we analyzed the patient profiles according to the cardiovascular outcomes assessment of the MitraClip percutaneous therapy for heart failure patients with functional mitral regurgitation trial (COAPT)-like profile if the following criteria were fulfilled: left ventricular (LV) ejection fraction ≥ 20%, LV end-systolic diameter ≤ 70 mm, tricuspid annular plane systolic excursion ≥ 15 mm, tricuspid regurgitation < severe, and systolic pulmonary artery pressure ≤ 70 mmHg [4].

### 2.3. Computed Tomographic (CT) Assessment

In patients considered for TMVR techniques, cardiac CT was conducted to assess the mitral valve morphologies before the heart team conference. The CT findings were retrospectively assessed by two dedicated physicians using 3mensio Structural Heart ver. 10.2 (3mensio Medical Imaging/Pie Medical Imaging, Netherlands). The mitral valve annulus and left ventricular outflow tract (LVOT) were assessed at the end-systolic and end-diastolic phases. The neo-LVOT area was simulated using suitable mitral valve prostheses according to intercommissural and anterior septal distances. We also measured the aortic mitral angle. The severity of mitral annular calcium was analyzed using a calcium thickness and calcium distribution based on a subjective semiquantitative approach, ranging from mild (fleck-like) to moderate (coalescing) to severe (bulky, protruding) [13]. In the present study, the non-eligible morphology for TMVR was defined as severe mitral annular calcification, aortic mitral angle <115°, neo-LVOT area <170 mm^2^, small LV chamber obliteration in the systolic phase, and an out-of-device size range simulated using intercommissural distance and anterior septal distance [13].

### 2.4. Outcome Measures

The primary outcome measure was the incidences of heart failure rehospitalization or mortality within one year. The procedural date was considered day 0 in patients undergoing a mitral valve treatment, whereas we defined the date of the institutional heart team conference as day 0 in patients with conservative management. All patients were followed up with routine outpatient visits and standardized telephone interviews with their families. The occurrence of heart failure was based on clinical diagnosis by the patients’ treating cardiologists or general practitioners. The secondary outcome measure was cardiovascular mortality within one year. The New York Heart Association (NYHA) functional class, severity of MR, and LV end-diastolic volume were assessed at baseline and at follow-up.

### 2.5. Statistical Analysis

Categorical variables are presented as absolute numbers and percentages. Continuous variables are described as the mean ± standard deviation if normally distributed and as the median and interquartile range (IQR) if not normally distributed.

To delve into the baseline characteristics of patients, we divided the cohort into two groups (i.e., intervention and medical therapy alone). Inter-group differences of continuous variables were tested with the Student’s *t*-test or Wilcoxon rank-sum test. Differences in categorical variables were examined by using the Chi-square test.

Next, we described the prevalence of each therapeutic option, successful device implantation, a reduction in MR to ≤2+, and mean mitral valve pressure gradient <5 mmHg at discharge. In contrast, detailed morphological characteristics predicting the procedural feasibility were listed in patients treated with medical therapy alone.

Finally, the clinical impact of mitral valve interventions was examined. A Kaplan–Meier method and log-rank test were applied to compare heart failure rehospitalization and all-cause mortality within one year. A Cox hazard regression analysis was conducted to investigate the association of the mitral valve intervention treatment with outcomes. The association was adjusted for sex, age, LV ejection fraction, estimated glomerular filtration rate, tricuspid annular plane systolic excursion, and the etiology of MR, based on previous knowledge [14,15,16,17]. Hazard ratios (HR) and 95% confidence intervals (95%CI) were calculated.

As a sensitivity analysis, patients treated with mitral valve interventions were further divided into two groups according to residual MR at discharge (i.e., MR ≤ 2+ and >2+). We conducted a Kaplan–Meier analysis and log-rank test to compare the outcomes between patients treated with medical therapy alone and patients according to residual MR at discharge. A two-tailed *p* < 0.05 was accepted as statistically significant. All statistical analyses were performed using JMP 14 version 14.3.0 (SAS Institute Inc, Cary, NC, USA).

## 3. Results

### 3.1. Study Participants

**Figure 1:**
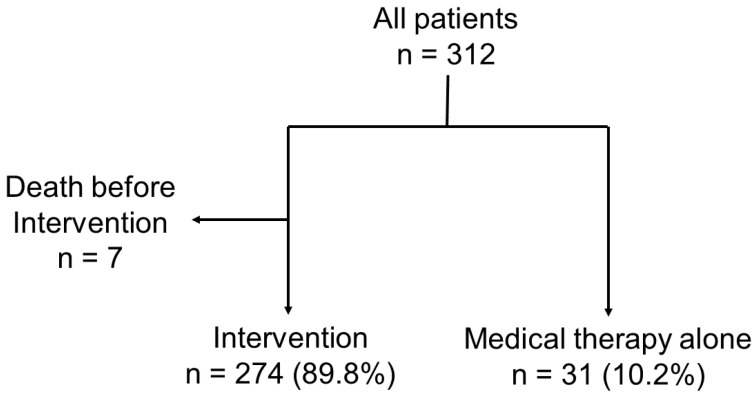
Study flow chart. The present study included 274 patients (89.8%), who underwent mitral valve interventions and 31 (10.2%) were treated with medical therapy alone.

A total of 305 patients were included in the present analysis (Figure 1). The study participants were 77.8 ± 8.3 years old and 52.5% female (Table 1). The majority of patients were highly symptomatic (NYHA functional class III/IV: 83.3%) and presented with a significant burden of comorbidities (coronary artery disease: 48.9%, atrial fibrillation: 80.3%, and eGFR: 38.9 mL/min/1.73 m^2^ [IQR 28.0, 48.9 mL/min/1.73 m^2^]), which translated into high surgical risk (EuroSCORE II: 6.75% [IQR 4.34, 10.29%]; the Society of Thoracic Surgeons predicted risk of mortality (STS PROM) score for mitral valve repair: 4.32% [IQR 2.71, 7.14%]; and STS PROM score for mitral valve replacement: 6.74% [IQR 4.16, 9.63%]). Most patients received a beta blocker (93.5%) and either an angiotensin-converting enzyme inhibitor, an angiotensin receptor blocker, or an angiotensin receptor–neprilysin inhibitor (71.5%). The mean LV ejection fraction was 52.6 ± 12.5%, and approximately half of the cohort exhibited secondary MR (*n* = 149: 48.9%). Among the patients with secondary MR, 42 (28.2%), a COAPT-like profile was exhibited, all of whom were treated with mitral valve interventions.

### 3.2. Patients with Mitral Valve Interventions

**Figure 2:**
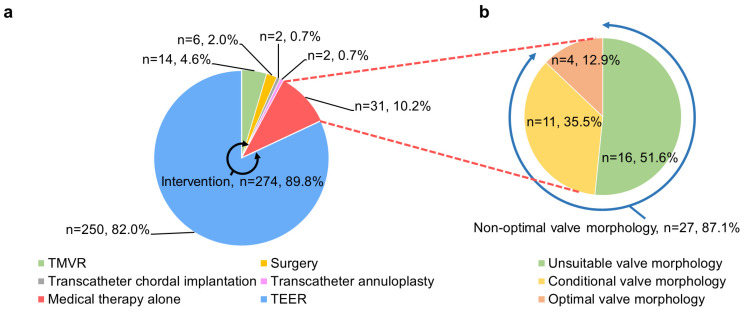
Contemporary therapeutic management of high surgical risk MR. Mitral valve intervention was performed in 89.8% of patients with high surgical risk, of which TEER was the most common approach. In contrast, 10.2% were treated with medical therapy alone (**a**). Among patients treated with medical therapy alone, 87.1% showed non-optimal valve morphology for TEER (**b**). Abbreviations: MR, mitral regurgitation; TEER, transcatheter edge-to-edge repair; and TMVR, transcatheter mitral valve replacement.

Of the 305 patients with MR ≥3+ and with a high surgical risk, 274 patients (89.8%) underwent mitral valve interventions, whereas 31 (10.2%) were treated with medical therapy alone. The baseline characteristics were comparable between the groups. The median duration from the interdisciplinary heart team conference to the procedure was 31 days (IQR 7, 45 days). Among the 274 mitral valve interventions, TEER was the most prevalent approach (*n* = 250, 82%), followed by TMVR (*n* = 14, 4.6%), mitral valve surgery (*n* = 6, 2.0%), transcatheter annuloplasty (*n* = 2, 0.7%), and transcatheter chordal implantation (*n* = 2, 0.7%) (Figure 2a). Of the six patients who received surgery, three patients underwent minimally invasive surgery.

Of the patients treated with mitral valve interventions, an MR reduction to ≤2+ at discharge was achieved in 233 patients (85.0%), and a mean mitral valve pressure gradient <5 mmHg at discharge was observed in 232 patients (84.7%). Device implantation was not achieved in 32 patients (11.7%) due to the following reasons: inadequate MR reduction (*n* = 21), increased mean mitral pressure gradient (*n* = 15), and/or technical complexity (*n* = 4).

The detailed procedural characteristics of TEER and TMVR are provided in Table 2 and Table 3. Among the 250 patients treated with TEER, successful device implantation was performed in 221 patients (88.4%), while no device implantation was observed in 29 patients (11.6%). An MR reduction to ≤2+ at discharge was achieved in 213 patients (85.2%). Among the 14 patients treated with TMVR, the Tendyne system was attempted in eight (57.2%) patients, the Highlife system in three (21.4%), and the Sapien3 system in three (21.4%). Treatment devices were successfully implanted in 12 (85.7%) patients, and a reduction in MR to ≤1+ was achieved in ten (71.4%) patients without any evidence of LVOT obstruction.

Additionally, two patients were treated with the Carillon transcatheter annuloplasty system, and an MR reduction to ≤2+ was achieved in all patients. Two patients were treated with transcatheter chordal implantation, in which the NeoChord system and Harpoon system were implanted in each patient. An MR reduction to ≤2+ at discharge was observed in the patient treated with the NeoChord system.

Re-interventions after their index procedure were performed in 22 out of 274 patients. Four patients underwent re-intervention during the index hospitalization for TEER. The reasons were single leaflet device attachment (*n* = 2), leaflet perforation during the procedure (*n* = 1), or no device implantation due to unsatisfactory MR reduction (*n* = 1). Another 18 patients underwent re-intervention within one year, of which transcatheter annuloplasty was performed in one patient at the index procedure, transcatheter chordal implantation in two patients, and TEER in fifteen patients. Out of 18 patients, TMVR was performed in nine patients at re-intervention, followed by TEER in six patients, whereas mitral valve surgery was conducted in three patients.

### 3.3. Patients with Medial Therapy Alone

**Figure 3:**
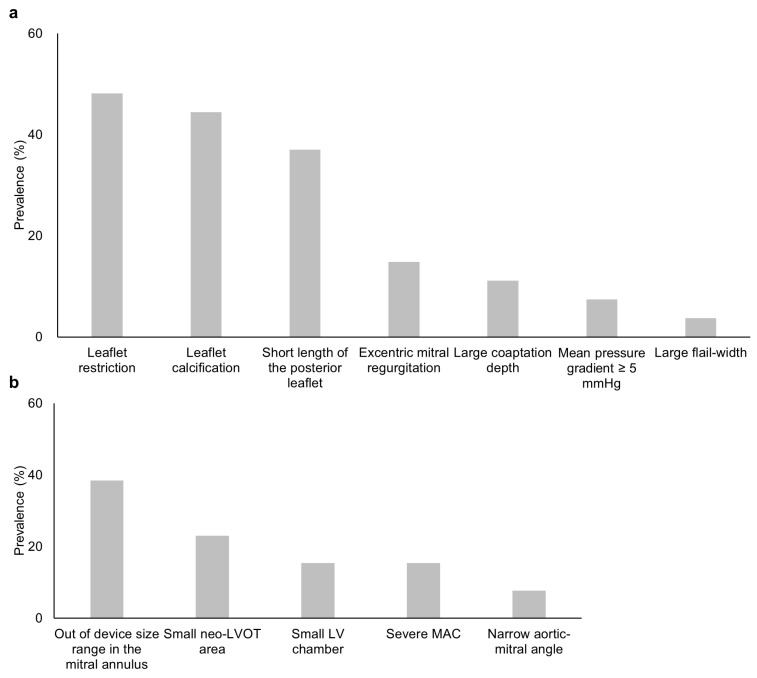
Details of non-optimal mitral valve morphology for TEER and TMVR. The details of non-optimal valve morphology were assessed for TEER by echocardiography (**a**). The details of CT assessment for TMVR are shown (**b**). Abbreviations: CT, computed tomography; LV, left ventricular; LVOT, left ventricular outflow tract; MAC, mitral annular calcification; TEER, transcatheter edge-to-edge mitral repair; and TMVR, transcatheter mitral valve replacement.

**Figure 4:**
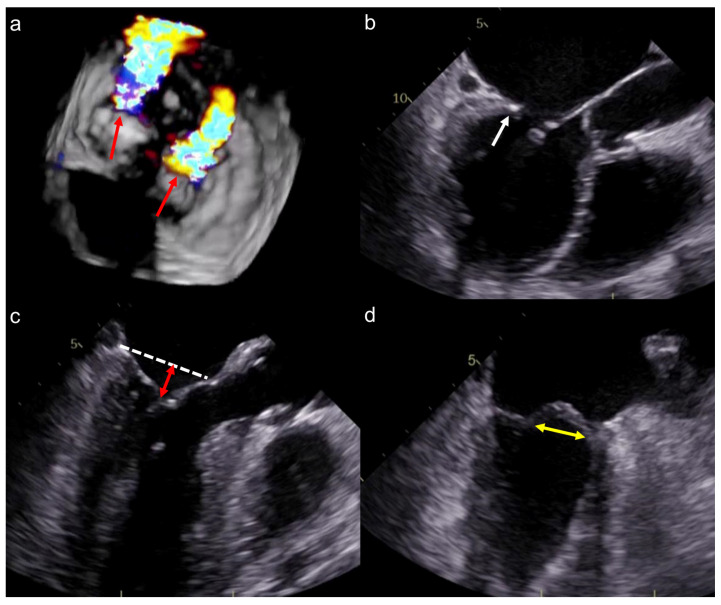
Representative cases of non-optimal valve morphologies for TEER. Red arrows indicate the MR jet in the lateral and medial segments (**a**). White arrow highlights the short length of the posterior leaflet with calcification (**b**). The red double-headed arrow depicts long coaptation height (**c**). The yellow double-headed arrow illustrates a long flail width (**d**). Abbreviations: MR, mitral regurgitation.

Among the 31 patients treated with medical therapy alone, 27 (87.1%) had at least one non-optimal anatomical feature for TEER (Figure 2b), according to the German criteria regarding echocardiographic assessments in mitral valve morphologies. The prevalence of each feature is listed in Figure 3a, and representative images are shown in Figure 4. For instance, severe leaflet restriction was observed in 13 (48.1%) patients. Elsewhere, leaflet calcification (12 patients, 44.4%), short length of the posterior leaflet (10 patients, 37.0%), eccentric MR (4 patients, 14.8%), large coaptation depth (3 patients, 11.1%), mean mitral valve pressure gradient ≥5 mmHg (2 patients 7.4%), and large flail width (1 patient, 3.7%) were also observed.

**Figure 5:**
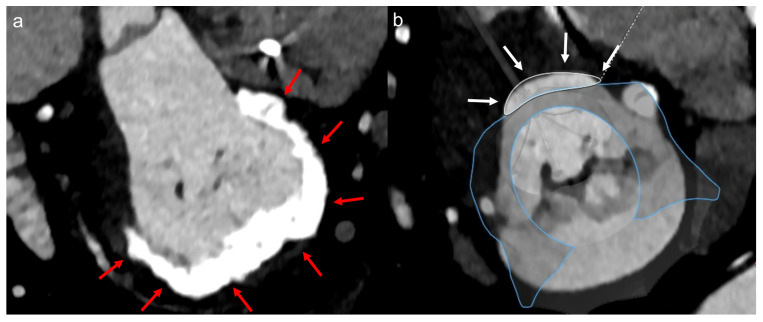
Representative cases of non-eligible valve morphologies for TMVR. Red arrows indicate severe mitral annular calcification (**a**). White curved outline and white arrows illustrate small neo-LVOT area. Blue curved line shape shows simulated transcatheter heart valve (**b**). Abbreviations: LVOT, left ventricular outflow tract and TMVR, transcatheter mitral valve replacement.

Cardiac CT assessment was available in 20 out of 31 patients, of whom 13 (65.0%) were deemed to have non-eligible morphology for TMVR or annuloplasty technique. The reasons were as follows: out of device size range in the mitral annulus (5 patients, 38.5%), small neo-LVOT area (3 patients, 23.1%), severe mitral annular calcification (2 patients, 15.4%), small LV chamber (2 patients, 15.4%), and narrow aortic-mitral angle (1 patient, 7.7%) (Figure 3b and Figure 5). Additional issues regarding the decision process were worsening for co-morbidities (e.g., psychosis, severely frail), symptom improvement, a decision by their referring physician, or the patient’s preference (Table 4).

### 3.4. Outcome of Patients

**Figure 6:**
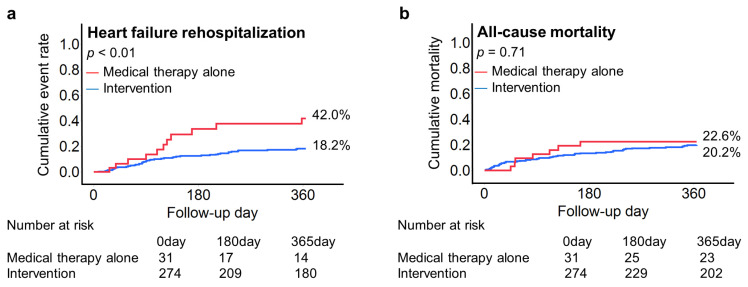
Heart failure rehospitalization and all-cause mortality within one year. The Kaplan–Meier curve depicts a lower cumulative event rate of heart failure rehospitalization in patients undergoing mitral valve interventions compared to patients treated with medical therapy (18.2% vs. 42.0%, *p* < 0.01) (**a**). In contrast, one-year all-cause mortality was similar between the groups (20.2% vs. 22.6%, *p* = 0.71) (**b**).

Within a median follow-up duration of 15.6 months (IQR 11.4, 21.4 months), 56 patients experienced heart failure rehospitalization (18.4%), and 61 died (20.0%) within one year. In the mitral valve intervention group, one-year cumulative heart failure rehospitalization was observed less often compared to the medical therapy alone group (18.2% vs. 42.0%, *p* < 0.01) (Figure 6a). In contrast, cumulative one-year mortality was similar between the two groups (20.2% vs. 22.6%, *p* = 0.71), as can be seen in Figure 6b. The findings were consistent in the multivariable Cox hazard regression model. Mitral valve intervention was associated with a decreased risk of heart failure rehospitalization within one year (HR 0.36, 95%CI 0.18–0.74, *p* < 0.01) (Table 5). In contrast, both managements were considered to be comparable for one-year all-cause mortality (mitral valve intervention vs. medical therapy alone: HR 1.02, 95%CI 0.36–2.89, *p* = 0.96) (Table 6). Similarly, cumulative one-year cardiovascular mortality was similar between the two groups (15.8% vs. 19.8%, *p* = 0.54).

**Figure 7:**
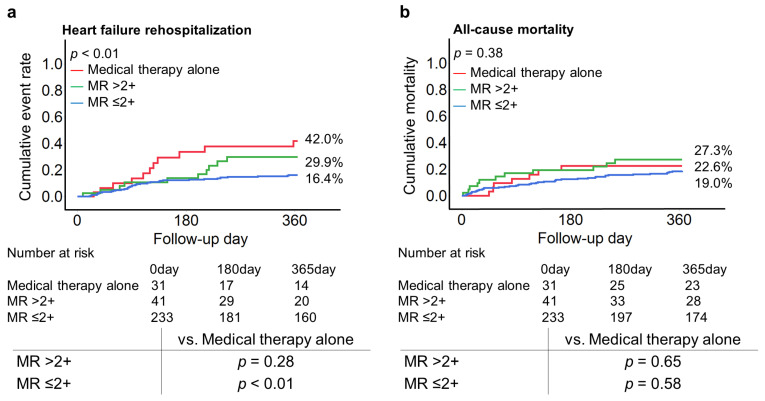
One-year heart failure hospitalization and all-cause mortality according to residual MR ≤ 2+, >2+ at discharge, and medical therapy alone. Patients with a reduction in MR to ≤2+ at discharge exhibited a lower event rate of heart failure rehospitalization compared to medical therapy alone (16.4% vs. 42.0%, *p* < 0.01) (**a**). However, patients with a residual MR > 2+ at discharge showed comparable outcomes with those treated with medical therapy alone (Heart failure rehospitalization: 29.9% vs. 42.0%, *p* = 0.28; all-cause mortality: 27.3% vs. 22.6%, *p* = 0.65) (**b**). Abbreviations: MR, mitral regurgitation.

In the sensitivity analysis, patients with a reduction in MR to ≤2+ at discharge exhibited a lower event rate of heart failure rehospitalization compared to medical therapy alone (16.4% vs. 42.0%, *p* < 0.01). However, patients with a residual MR > 2+ at discharge showed comparable outcomes with those treated with medical therapy alone (Heart failure rehospitalization: 29.9% vs. 42.0%, *p* = 0.28; all-cause mortality: 27.3% vs. 22.6%, *p* = 0.65) (Figure 7).

**Figure 8:**
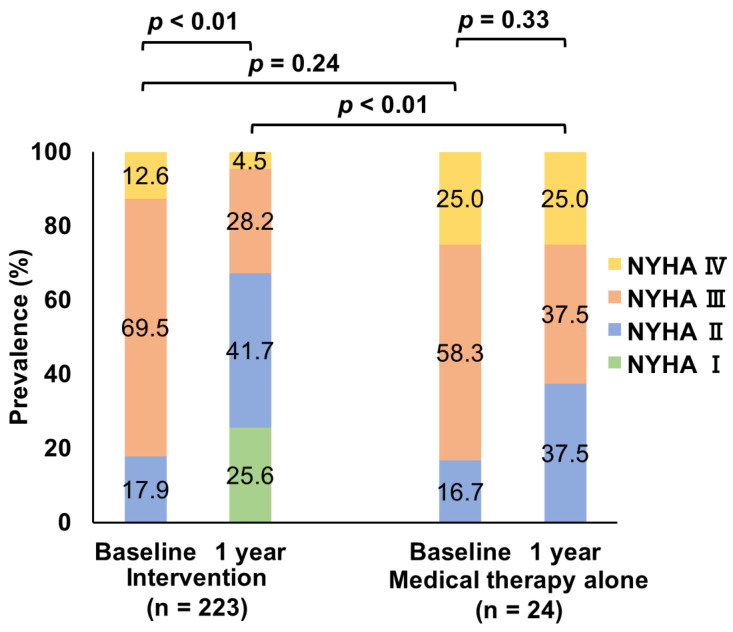
Changes in NYHA functional class. Mitral valve intervention was associated with an improvement in the NYHA functional class from baseline to one-year follow-up (*p* < 0.01). Patients undergoing mitral valve interventions had lower NYHA functional scales compared to medical therapy alone within one-year follow-up (*p* < 0.01). Abbreviations: NYHA, New York Heart Association.

The NYHA functional class at one-year follow-up was available in 247 patients. Patients who underwent mitral valve interventions showed an improvement in symptoms from baseline to one-year follow-up (*p* < 0.01), whereas there was no significant change in patients treated with medical therapy alone (*p* = 0.33). Accordingly, patients who underwent mitral valve interventions had lower NYHA functional scales compared to those treated with medical therapy alone (*p* < 0.01) (Figure 8).

**Figure 9:**
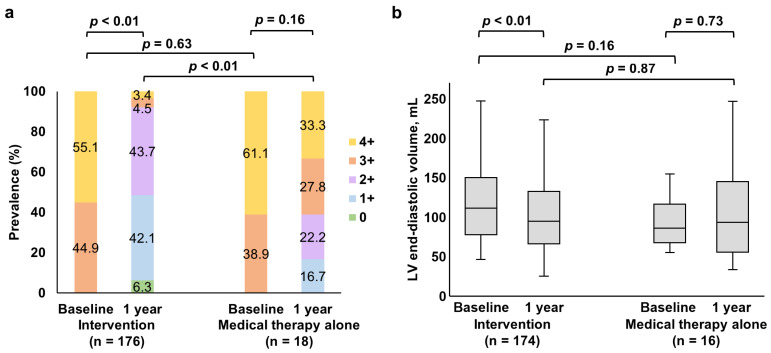
Changes in severity of MR and LV end-diastolic volume. At one year, patients undergoing mitral valve interventions had a lower severity of MR compared to medical therapy alone (*p* < 0.01) (**a**). Mitral valve intervention was associated with decreased LV end-diastolic volume from baseline to one-year follow-up (*p* < 0.01). LV end-diastolic volume within one year follow-up was similar between the two groups (94.9 mL [66.3, 132.3] vs. 93.3 mL [55.6, 144.7], *p* = 0.87) (**b**). Abbreviations: LV, left ventricular and MR, mitral regurgitation.

Echocardiography at a one-year follow-up was available in 194 patients. At one year, patients undergoing mitral valve interventions had lower MR grades compared to medical therapy alone (*p* < 0.01) (Figure 9a). Additionally, LV end-diastolic volume decreased from baseline to one-year follow-up in patients treated with mitral valve interventions (*p* < 0.01). In contrast, there was no significant change in LV end-diastolic volume in the medical therapy group (*p* = 0.73) (Figure 9b). The LV end-diastolic volume at the one-year follow-up was similar between the two groups (94.9 mL [IQR 66.3, 132.3 mL] vs. 93.3 mL [IQR 55.6, 144.7 mL], *p* = 0.87).

## 4. Discussion

We aimed to assess the therapeutic strategy and clinical outcome of patients with MR and high surgical risk in the current clinical practice. The main findings can be summarized as follows:

1. Mitral valve intervention was performed in 89.8% of MR patients with high surgical risk. TEER was the most common approach (82.0% of overall), followed by TMVR (4.6%), surgery (2.0%), transcatheter annuloplasty (0.7%), and transcatheter chordal implantation (0.7%).
2. Among patients treated with medical therapy alone, 87.1% of these patients showed non-optimal valve morphology for TEER, and 65.0% were deemed as having non-eligible morphology for TMVR.
3. Mitral valve intervention was associated with a decreased risk of rehospitalization due to heart failure compared to medical therapy alone.

The development of transcatheter therapies has revolutionized the landscape of MR treatment in patients with high surgical risk [4]. TEER has become a widely accepted alternative to surgery, showing its safety and efficacy on clinical prognosis and its functional status [4,5]. While TEER was performed as the most frequent therapeutic approach in the present study, other transcatheter technologies have also shown remarkable progress, and treatment options for MR are expanding [6,7,8]. The development of these complementary device technologies may offer physicians a wide array of potential treatment options for optimal individual therapies.

The present study participants were elderly, highly symptomatic, and had a large burden of comorbidities, which aligned with the previous studies investigating patients with MR and high surgical risk [8,18,19]. In the present analysis, we found that 89.8% of patients underwent mitral valve interventions. Device successful implantation was observed in 88.3%, and an MR reduction to ≤2+ at discharge was observed in 85.0% after the mitral valve interventions, which was slightly lower compared to a previous study investigating TEER for MR [18,19]. More specifically, TEER seemed to show a 12.8% (32 patients, of whom 29 implanted no device and three required re-intervention) of device implantation failure during the index hospitalization. This may be attributable to the inclusion of all-comer patients of high surgical risk MR in the present analysis. For instance, the prevalence of a prior history of mitral valve treatment (8.4% in the present cohort) was higher than those in previous studies (up to 2.5%) [18]; therefore, this might contribute to the rate of residual MR. Moreover, a potential explanation is related to institutional therapeutic strategies. Device implantation with a suboptimal MR reduction is discouraged in our practice, and secondary interventional or surgical alternatives are always considered if an adequate procedural result is not estimated during the procedure. Leaving a device in the mitral position without an adequate MR reduction may be associated with recurrent MR [20] and may limit the potential of device therapies in the future. In the present cohort, among the 32 patients who did not receive any device implantation at their initial intervention, 16 underwent re-intervention within one year. The variety of available technologies enables physicians not to stick with a single therapeutic option but to choose alternative treatments to gain optimal procedural results.

Despite the multiple device options in the current clinical practice, 10.2% of present study participants were treated with medical therapy alone. In general, reasons for conservative therapy are likely to be multifactorial. One of the major factors for the decision making is the mitral valve morphology, aiming to achieve a safe and effective MR reduction by applying transcatheter technologies. Complex anatomy is associated with inappropriate procedural results and prognosis after TEER [20]. In the present study, 87.1% of patients treated with medical therapy alone showed non-optimal valve morphology for TEER according to the German criteria [12]. For those patients, TMVR may be an attractive solution [8]. Wild MG et al., reported that an MR reduction to 1+ or less was achieved in 100% of patients who underwent TMVR, even though most patients showed challenging anatomies for TEER. However, LVOT obstruction after TMVR is a major concern associated with mortality [21]. According to the present analysis of consecutive MR patients, most patients (65.0%) were deemed to be non-eligible for TMVR. In line with our findings, previous studies have reported that up to 75% of patients with MR are considered to have non-suitable mitral valve morphology for TEER and TMVR in their screening phase [22,23,24,25]. The other limitations of TMVR techniques are (1) the need for preprocedural contrast-enhanced CT scans and (2) the overall complexity of currently available devices.

Transcatheter chordal implantation is an alternative option for primary MR, and transcatheter annuloplasty is for secondary MR. Both technologies remain as alternative options to TEER thus far [26,27,28]. However, they have been reported to offer several positive advantages for the treatment of MR. The transventricular chordal implantation mimics the surgical repair technique in degenerative MR and can fully restore the mitral valve function without the risk of mitral stenosis; it also allows the chance of standard repair in the case of failure. The ventricular approach remains at a disadvantage as it is more invasive than percutaneous approaches, but this technology will soon be able to be applied to transseptal approaches [29,30,31]. For the transcatheter annuloplasty system, one of the most important advantages is that the possibility of almost all interventions remains open after the procedure. Although the dedicated preprocedural evaluation is essential, such as the proximity to coronary sinus or coronary arteries [32,33], this technology will be highlighted in the near future, once its safety and efficacy have been proven. Therefore, advances in device iterations with better delivery systems and even less invasive approaches will be milestones to broaden the scope of transcatheter therapy, thereby improving the clinical outcomes of patients with MR.

Regarding clinical outcomes, patients treated with mitral valve interventions had a lower rate of heart failure rehospitalization, a lower NYHA functional class, and a lower severity of MR within one year compared to patients treated with medical therapy alone. Our findings align with the COAPT trial [4], which showed a marked reduction in the risk of rehospitalization and an improved NYHA functional class after TEER over medical therapy alone. In contrast, all-cause mortality was comparable between both managements in the present study, which may be attributable to a limited sample size and follow-up period. Alternatively, the development of medical therapies in the current clinical practice might have improved patient outcomes, given that the rate of adherence to guideline-directed optimal medical therapies seemed to be higher compared to earlier observational studies [18]. Another possible explanation might be that most patients with secondary MR in the intervention group had a non-COAPT-like profile. Adamo et al., reported that patients with non-COAPT-like profiles had higher mortality rates than those with COAPT-like profiles with secondary MR [34]. With that in mind, our findings might imply that there are patients in whom mitral valve interventions are useless in improving clinical prognosis. Of note, patients with a residual MR of >2+ after mitral valve interventions seemed to be numerically higher in terms of mortality compared to medical therapy alone, reinforcing the importance of the safety and feasibility of applying a mitral valve intervention. Therefore, further advances in device technologies would be milestones, leading to the potential to broaden the scope of transcatheter therapy.

## 5. Study Limitations

Several limitations should be acknowledged. First, the present study is a retrospective analysis based on a single-center observational cohort. However, the baseline characteristics of the study participants were similar to previous studies [8,18,19]. Second, there might be immortal time bias in patients treated with mitral valve interventions since the patients were retrospectively assigned to each cohort. However, in the present analysis, the follow-up start date for the mitral intervention and medical therapy alone were set separately, which should, at least partially, address the inherent potential immortal time bias. Third, the role of center and operator experience cannot be ignored, given the association between increased experiences and procedural outcomes of transcatheter mitral valve treatments [35]. Fourth, due to the limited sample size, we did not perform a stratification analysis according to the etiology of MR. However, the association of mitral valve treatment with outcomes was adjusted by baseline covariates, including the MR etiology. Moreover, we provided detailed information on the mitral valve morphologies of our collective.

## 6. Conclusions

In the present study, we found that most cases were treated with mitral valve interventions in the contemporary management of patients with MR and high surgical risk. Of these, TEER was currently considered a standard of care, whereas other transcatheter approaches could provide complementary options. Non-neglectable number of patients were treated with medical therapy alone due to challenging anatomy for modern interventional technologies. A mitral valve intervention was associated with a lower risk of heart failure rehospitalization and improved NYHA functional class. Hence, further development of device technologies may be mandatory to address various mitral valve morphologies and could improve procedural outcomes and patient prognosis.

## Figures and Tables

**Table 1:** Patient characteristics.

	All Patients *n* = 305	Medical Therapy Alone *n* = 31	Intervention *n* = 274	*p*-Value
**Baseline demographics**				
Age, years	77.8 ± 8.3	79.2 ± 8.2	77.7 ± 8.3	0.32
Sex male, *n* (%)	145 (47.5)	14 (45.2)	131 (47.8)	0.78
Coronary artery disease, *n* (%)	149 (48.9)	15 (48.4)	134 (48.9)	0.96
Prior PCI, *n* (%)	107 (35.1)	11 (35.5)	96 (35.0)	0.96
Prior CABG, *n* (%)	39 (12.8)	4 (12.9)	35 (12.8)	0.98
Prior mitral valve therapy, *n* (%)	25 (8.2)	4 (12.9)	21 (7.7)	0.30
Surgery, *n* (%)	6 (2.0)	1 (3.2)	5 (1.8)	0.48
Transcatheter treatment, *n* (%)	21 (6.9)	4 (12.9)	17 (6.2)	0.25
Atrial fibrillation, *n* (%)	245 (80.3)	26 (83.9)	219 (79.9)	0.60
NYHA III or IV, *n* (%)	254 (83.3)	25 (80.7)	229 (83.6)	0.68
EuroSCORE II, %	6.75 (4.34, 10.29)	7.85 (5.45, 10.59)	6.46 (4.21, 10.12)	0.13
STS PROM score for MV repair, %	4.32 (2.71, 7.14)	5.89 (3.09, 8.93)	4.27 (2.67, 6.89)	0.11
STS PROM score for MV replacement, %	6.74 (4.16, 9.63)	7.62 (5.20, 10.54)	6.65 (4.57, 9.62)	0.39
eGFR, mL/min/1.73 m^2^	38.9 (28.0, 48.9)	35.8 (23.7, 51.4)	39.0 (28.6, 48.4)	0.95
NT pro-BNP, pg/mL	2258 (1318, 4118)	4078 (1212, 8311)	2200 (1320, 4083)	0.19
**Medical treatment**				
Beta blockers, *n* (%)	262 (85.9)	29 (93.6)	233 (85.0)	0.28
ACE-I, ARB, ARNI, *n* (%)	218 (71.5)	22 (71.0)	196 (71.5)	0.95
MRA, *n* (%)	106 (34.8)	6 (19.4)	100 (36.5)	0.06
Diuretics, *n* (%)	178 (91.2)	28 (90.3)	250 (91.2)	0.75
**Echocardiographic parameters**				
LV ejection fraction, %	52.3 ± 12.4	49.5 ± 12.0	53.0 ± 12.6	0.15
LV end-diastolic volume, mL	95.8 (68.6, 134.3)	87.7 (78.5, 133.8)	107.5 (74.8, 147.5)	0.43
LV end-systolic volume, mL	42.9 (27.8, 68.2)	48.2 (30.1, 70.2)	45.1 (30.4, 70.1)	0.76
Left atrial volume, mL	90.0 (67.8, 119.4)	79.0 (52.0, 123.5)	90.0 (69.2, 118.4)	0.29
Severity of MR, *n* (%)				0.46
3+	137 (44.9)	12 (39.7)	125 (45.4)	
4+	168 (55.1)	19 (61.3)	149 (54.4)	
Etiology of MR, *n* (%)				0.39
Primary	131 (43.0)	15 (48.4)	116 (42.3)	
Secondary	149 (48.9)	12 (38.7)	137 (50.0)	
Recurrent	25 (8.2)	4 (12.9)	21 (7.7)	
Effective regurgitant orifice area, cm^2^	0.3 (0.2, 0.4)	0.37 (0.23, 0.44)	0.3 (0.2, 0.4)	0.10
Regurgitation volume, mL	49.7 (35.0, 65.0)	47.3 (40.3, 57.5)	50.0 (35.0, 65.0)	0.50
Tricuspid regurgitation ≥ moderate	256 (80.5)	25 (80.7)	221 (80.7)	0.99
sPAP, mmHg	40.6 ± 13.6	37.2 ± 11.4	41.0 ± 13.8	0.16
TAPSE, mm	18.9 ± 5.4	18.1 ± 4.4	19.0 ± 5.5	0.38
Mean MV pressure gradient, mmHg	1.9 (1.2, 3.1)	3.0 (1.6, 3.8)	1.9 (1.2, 3.1)	0.20

Abbreviations: ACE-I, angiotensin-converting enzyme inhibitor; ARB, angiotensin receptor blocker; ARNI, angiotensin receptor–neprilysin inhibitor; CABG, coronary artery bypass graft; eGFR, estimated glomerular filtration rate; LV, left ventricular; MRA, mineralocorticoid receptor antagonists; MV, mitral valve; NYHA, New York Heart Association; NT-pro BNP, N-terminal prohormone of brain natriuretic peptide; PCI, percutaneous coronary intervention; sPAP, systolic pulmonary artery pressure; STS PROM, Society of Thoracic Surgeons predicted risk of mortality; and TAPSE, tricuspid annular plane systolic excursion.

**Table 2:** Procedural characteristics of TEER.

	*n* = 250
Type of device system, *n* (%)	
MitraClip	228 (91.2)
PASCAL	22 (8.8)
Number of clips implanted, *n* (%)	
0	29 (11.6)
1	135 (54.0)
2	77 (30.8)
3	9 (3.6)
Procedural time, min	66 (49, 93)
Mean mitral valve pressure gradient at discharge, mmHg	3.3 (2.3, 4.4)
Mean mitral valve pressure gradient <5 mmHg at discharge, *n* (%)	214 (85.6)
Severity of MR ≤ 2+ at discharge, *n* (%)	213 (86.4%)

Abbreviations: MR, mitral regurgitation and TEER, transcatheter edge-to-edge repair.

**Table 3:** Procedural characteristics of TMVR.

	*n* = 14
Type of device system, *n* (%)	
Tendyne	8 (57.2)
Highlife	3 (21.4)
Sapien3	3 (21.4)
No device implantation, *n* (%)	2 (14.3)
Procedural time, min	138 (82, 206)
Mean mitral valve pressure gradient at discharge, mmHg	4.1 (2.8, 5.4)
Mean mitral valve pressure gradient <5 mmHg at discharge, *n* (%)	10 (71.4)
Severity of MR ≤ 1+ at discharge, *n* (%)	10 (71.4%)
LVOT mean pressure gradient at discharge, mmHg	2.7 ± 1.7
LVOT obstruction at discharge, *n* (%)	0 (0)

Abbreviations: MR, mitral regurgitation and TMVR, transcatheter mitral valve replacement.

**Table 4:** Reasons for medical therapy alone.

Non-optimal anatomy for transcatheter mitral valve therapy
Improved symptoms before procedure
Decreased severity in MR before procedure
Worsening of general condition or comorbidity
Severe frailty
Gastrointestinal hemorrhage
Decision by the referring physician
Patient’s preference

Abbreviations: MR, mitral regurgitation.

**Table 5:** Association of mitral valve intervention with one-year heart failure rehospitalization.

	Univariable Analysis			Multivariable Analysis		
	HR	94%CI	*p*-Value	HR	95%CI	*p*-Value
Sex male	1.72	1.01–2.94	0.04	2.16	1.18–3.93	0.01
Age (per 1 year increase)	0.99	0.96–1.03	0.64	1.00	0.97–1.04	0.83
Mitral valve intervention	0.39	0.20–0.76	<0.01	0.36	0.18–0.74	<0.01
eGFR (per 1 mL/min/1.73 m^2^ increase)	0.97	0.96–0.99	<0.01	0.97	0.96–0.99	<0.01
LV ejection fraction (per 1% increase)	0.98	0.96–1.02	0.08	1.01	0.98–1.04	0.50
TAPSE <15 mm	1.90	1.05–3.43	0.03	1.78	0.95–3.32	0.07
Secondary MR	1.15	0.68–1.94	0.61	1.29	0.70–2.40	0.41

Abbreviations: eGFR, estimated glomerular filtration rate; LV, left ventricular; MR, mitral regurgitation; and TAPSE, tricuspid annular plane systolic excursion.

**Table 6:** Association of mitral valve intervention with one-year all-cause mortality.

	Univariable Analysis			Multivariable Analysis		
	HR	95%CI	*p*-Value	HR	95%CI	*p*-Value
Sex male	1.55	0.94–2.58	0.09	1.94	1.10–3.41	0.02
Age (per 1 year increase)	0.97	0.95–1.00	0.05	1.00	0.96–1.03	0.82
Mitral valve Intervention	0.86	0.39–1.89	0.71	0.74	0.31–1.77	0.74
eGFR (per 1 mL/min/1.73 m^2^ increase)	0.98	0.96–0.99	<0.01	0.98	0.96–0.99	<0.01
LV ejection fraction (per 1% increase)	0.99	0.98–1.02	0.62	1.03	1.00–1.05	0.04
TAPSE < 15 mm	2.58	1.51–4.41	<0.01	2.59	1.47–4.57	<0.01
Secondary MR	1.24	0.75–2.05	0.41	1.67	0.93–3.01	0.09

Abbreviations: eGFR, estimated glomerular filtration rate; LV, left ventricular; MR, mitral regurgitation; and TAPSE, tricuspid annular plane systolic excursion.

## Data Availability

Data can be obtained from the corresponding author upon reasonable request.

## References

[1] Enriquez-Sarano M., Akins C.W., Vahanian A. (2009). Mitral regurgitation. Lancet.

[2] Dziadzko V., Clavel M.A., Dziadzko M., Medina-Inojosa J.R., Michelena H., Maalouf J., Nkomo V., Thapa P., Enriquez-Sarano M. (2018). Outcome and undertreatment of mitral regurgitation: A community cohort study. Lancet.

[3] Gheorghe L.L., Mobasseri S., Agricola E., Wang D.D., Milla F., Swaans M., Pandis D., Adams D.H., Yadav P., Sievert H. (2021). Imaging for Native Mitral Valve Surgical and Transcatheter Interventions. JACC Cardiovasc. Imag..

[4] Stone G.W., Lindenfeld J., Abraham W.T., Kar S., Lim D.S., Mishell J.M., Whisenant B., Grayburn P.A., Rinaldi M., Kapadia S.R. (2018). Transcatheter Mitral-Valve Repair in Patients with Heart Failure. N. Engl. J. Med..

[5] Lim D.S., Reynolds M.R., Feldman T., Kar S., Herrmann H.C., Wang A., Whitlow P.L., Gray W.A., Grayburn P., Mack M.J. (2014). Improved functional status and quality of life in prohibitive surgical risk patients with degenerative mitral regurgitation after transcatheter mitral valve repair. J. Am. Coll. Cardiol..

[6] Mack M., Carroll J.D., Thourani V., Vemulapalli S., Squiers J., Manandhar P., Deeb G.M., Batchelor W., Herrmann H.C., Cohen D.J. (2021). Transcatheter Mitral Valve Therapy in the United States: A Report From the STS-ACC TVT Registry. J. Am. Coll. Cardiol..

[7] Muller D.W.M., Sorajja P., Duncan A., Bethea B., Dahle G., Grayburn P., Babaliaros V., Guerrero M., Thourani V.H., Bedogni F. (2021). 2-Year Outcomes of Transcatheter Mitral Valve Replacement in Patients With Severe Symptomatic Mitral Regurgitation. J. Am. Coll. Cardiol..

[8] Wild M.G., Kreidel F., Hell M.M., Praz F., Mach M., Adam M., Reineke D., Ruge H., Ludwig S., Conradi L. (2022). Transapical mitral valve implantation for treatment of symptomatic mitral valve disease: A real-world multicentre experience. Eur. J. Heart Fail..

[9] Stone G.W., Vahanian A.S., Adams D.H., Abraham W.T., Borer J.S., Bax J.J., Schofer J., Cutlip D.E., Krucoff M.W., Blackstone E.H. (2015). Clinical Trial Design Principles and Endpoint Definitions for Transcatheter Mitral Valve Repair and Replacement: Part 1: Clinical Trial Design Principles: A Consensus Document From the Mitral Valve Academic Research Consortium. J. Am. Coll. Cardiol..

[10] Vahanian A., Beyersdorf F., Praz F., Milojevic M., Baldus S., Bauersachs J., Capodanno D., Conradi L., De Bonis M., De Paulis R. (2022). 2021 ESC/EACTS Guidelines for the management of valvular heart disease. Eur. Heart J..

[11] Zoghbi W.A., Adams D., Bonow R.O., Enriquez-Sarano M., Foster E., Grayburn P.A., Hahn R.T., Han Y., Hung J., Lang R.M. (2017). Recommendations for Noninvasive Evaluation of Native Valvular Regurgitation: A Report from the American Society of Echocardiography Developed in Collaboration with the Society for Cardiovascular Magnetic Resonance. J. Am. Soc. Echocardiogr..

[12] Boekstegers P., Hausleiter J., Baldus S., von Bardeleben R.S., Beucher H., Butter C., Franzen O., Hoffmann R., Ince H., Kuck K.H. (2014). Percutaneous interventional mitral regurgitation treatment using the Mitra-Clip system. Clin. Res. Cardiol..

[13] Reid A., Ben Zekry S., Turaga M., Tarazi S., Bax J.J., Wang D.D., Piazza N., Bapat V.N., Ihdayhid A.R., Cavalcante J.L. (2021). Neo-LVOT and Transcatheter Mitral Valve Replacement: Expert Recommendations. JACC Cardiovasc. Imag..

[14] Ya’Qoub L., Gad M., Faza N.N., Kunkel K.J., Ya’acoub R., Villablanca P., Bagur R., Alasnag M., Eng M., Elgendy I.Y. (2022). Sex differences in outcomes of transcatheter edge-to-edge repair with MitraClip: A meta-analysis. Catheter. Cardiovasc. Interv..

[15] Kessler M., Seeger J., Muche R., Wohrle J., Rottbauer W., Markovic S. (2019). Predictors of rehospitalization after percutaneous edge-to-edge mitral valve repair by MitraClip implantation. Eur. J. Heart Fail..

[16] Sawalha K., Al-Akchar M., Ibrahim A., Buhnerkempe M., Koester C., Salih M., Bhattarai M., Tandan N., Bhatt D.L., Hafiz A.M. (2021). Impact of chronic kidney disease on in-hospital outcomes and readmission rate after edge-to-edge transcatheter mitral valve repair. Catheter. Cardiovasc. Interv..

[17] Kaneko H., Neuss M., Weissenborn J., Butter C. (2016). Prognostic Significance of Right Ventricular Dysfunction in Patients With Functional Mitral Regurgitation Undergoing MitraClip. Am. J. Cardiol..

[18] Bedogni F., Popolo Rubbio A., Grasso C., Adamo M., Denti P., Giordano A., Tusa M., Bianchi G., De Marco F., Bartorelli A.L. (2021). Italian Society of Interventional Cardiology (GIse) registry Of Transcatheter treatment of mitral valve regurgitaTiOn (GIOTTO): Impact of valve disease aetiology and residual mitral regurgitation after MitraClip implantation. Eur. J. Heart Fail..

[19] Armijo G., Estevez-Loureiro R., Carrasco-Chinchilla F., Arzamendi D., Fernandez-Vazquez F., Jimenez-Quevedo P., Freixa X., Pascual I., Serrador A.M., Mesa D. (2020). Acute Kidney Injury After Percutaneous Edge-to-Edge Mitral Repair. J. Am. Coll. Cardiol..

[20] Sugiura A., Kavsur R., Spieker M., Iliadis C., Goto T., Ozturk C., Weber M., Tabata N., Zimmer S., Sinning J.M. (2022). Recurrent Mitral Regurgitation After MitraClip: Predictive Factors, Morphology, and Clinical Implication. Circ. Cardiovasc. Interv..

[21] Russo G., Gennari M., Gavazzoni M., Pedicino D., Pozzoli A., Taramasso M., Maisano F. (2021). Transcatheter Mitral Valve Implantation: Current Status and Future Perspectives. Circ. Cardiovasc. Interv..

[22] Forrestal B.J., Khan J.M., Torguson R., Case B.C., Safren L., Nasher N., Reddin G., Satler L., Ben-Dor I., Shults C. (2021). Reasons for Screen Failure for Transcatheter Mitral Valve Repair and Replacement. Am. J. Cardiol..

[23] Niikura H., Gossl M., Kshettry V., Olson S., Sun B., Askew J., Stanberry L., Garberich R., Tang L., Lesser J. (2019). Causes and Clinical Outcomes of Patients Who Are Ineligible for Transcatheter Mitral Valve Replacement. JACC Cardiovasc. Interv..

[24] Mangieri A., Melillo F., Montalto C., Denti P., Praz F., Sala A., Winkel M.G., Taramasso M., Tagliari A.P., Fam N.P. (2022). Management and Outcome of Failed Percutaneous Edge-to-Edge Mitral Valve Plasty: Insight From an International Registry. JACC Cardiovasc. Interv..

[25] Urena M., Vahanian A., Sondergaard L. (2018). Patient selection for transcatheter mitral valve implantation: Why is it so hard to find patients?. EuroIntervention.

[26] Siminiak T., Wu J.C., Haude M., Hoppe U.C., Sadowski J., Lipiecki J., Fajadet J., Shah A.M., Feldman T., Kaye D.M. (2012). Treatment of functional mitral regurgitation by percutaneous annuloplasty: Results of the TITAN Trial. Eur. J. Heart Fail..

[27] Gammie J.S., Bartus K., Gackowski A., D’Ambra M.N., Szymanski P., Bilewska A., Kusmierczyk M., Kapelak B., Rzucidlo-Resil J., Moat N. (2018). Beating-Heart Mitral Valve Repair Using a Novel ePTFE Cordal Implantation Device: A Prospective Trial. J. Am. Coll. Cardiol..

[28] Vairo A., Gaiero L., Marro M., Russo C., Bolognesi M., Soro P., Gallone G., Fioravanti F., Desalvo P., D’Ascenzo F. (2023). New Echocardiographic Parameters Predicting Successful Trans-Ventricular Beating-Heart Mitral Valve Repair with Neochordae at 3 Years: Monocentric Retrospective Study. J. Clin. Med..

[29] Latib A., Ho E.C., Scotti A., Modine T., Shaburishvili T., Zirakashvili T., Von Bardeleben R.S., Chitwood W.R. (2022). First-in-Human Transseptal Transcatheter Mitral Chordal Repair. JACC Cardiovasc. Interv..

[30] Colli A., Manzan E., Aidietis A., Rucinskas K., Bizzotto E., Besola L., Pradegan N., Pittarello D., Janusauskas V., Zakarkaite D. (2018). An early European experience with transapical off-pump mitral valve repair with NeoChord implantation. Eur. J. Cardiothorac. Surg..

[31] Colli A., Manzan E., Aidietis A., Rucinskas K., Bizzotto E., Besola L., Pradegan N., Pittarello D., Janusauskas V., Zakarkaite D. (2019). Corrigendum to: ‘An early European experience with transapical off-pump mitral valve repair with NeoChord implantation [Eur J Cardiothorac Surg 2018;54:460-6]. Eur. J. Cardiothorac. Surg..

[32] De Backer O., Wong I., Taramasso M., Maisano F., Franzen O., Sondergaard L. (2021). Transcatheter mitral valve repair: An overview of current and future devices. Open Heart.

[33] Koell B., Kalbacher D., Lubos E. (2021). Current devices and interventions in mitral regurgitation. Herz.

[34] Adamo M., Fiorelli F., Melica B., D’Ortona R., Lupi L., Giannini C., Silva G., Fiorina C., Branca L., Chiari E. (2021). COAPT-Like Profile Predicts Long-Term Outcomes in Patients With Secondary Mitral Regurgitation Undergoing MitraClip Implantation. JACC Cardiovasc. Interv..

[35] Eleid M.F., Reeder G.S., Malouf J.F., Lennon R.J., Pislaru S.V., Nkomo V.T., Rihal C.S. (2016). The Learning Curve for Transcatheter Mitral Valve Repair With MitraClip. J. Interv. Cardiol..

